# Lesser sac herniation: a rare cause of acute abdomen and bowel perforation

**DOI:** 10.1259/bjrcr.20150501

**Published:** 2016-06-29

**Authors:** Asad N Tamimi, Samuel J Withey, Sami Ullah Khan

**Affiliations:** ^1^Department of Radiology, Basildon and Thurrock University Hospital, Basildon, UK; ^2^Department of Radiology, King’s College, London, UK

## Abstract

Lesser sac herniation is a rare phenomenon, where the bowel protrudes through the epiploic foramen into the lesser sac. We describe the case of a 55-year-old male who presented with acute abdominal pain and in whose case the subtle findings of lesser sac herniation were missed during CT scan reporting. Re-review of the images after the patient’s condition deteriorated found evidence of herniation, and re-scanning at that point demonstrated progression of the herniation, with bowel obstruction and perforation. The findings of lesser sac herniation complicated by basculetype caecal volvulus were confirmed during laparotomy. The patient underwent right hemicolectomy with primary ileocolic anastomosis.

## Clinical presentation

A 55-year-old male presented to the local emergency department with abdominal pain. He complained of central “crampy” pain radiating to his back, associated with bilious vomiting. His bowels, previously opening daily, had not opened for the past 48 h.

On examination, the patient was tachycardic (heart rate 105), hypotensive (blood pressure 96/67 mmHg), afebrile and maintaining good oxygen saturations on air. On inspection, he had a raised body mass index. Cardiovascular and respiratory examinations were normal. Abdominal palpation revealed epigastric and right hypochondrium tenderness but no signs of peritonism. Rectal examination was unremarkable. Prior to this, the patient was fit and well. He reported no past surgical or medical history apart from hypertension, for which he was taking amlodipine. The patient’s alcohol intake was 21 units per week and he was an ex-smoker, with a 12 pack-year history.

## Differential diagnoses

- Leaking abdominal aortic aneurysm.
- Acute pancreatitis.
- Ischaemic bowel.

## Investigations

Blood results: mild leukocytosis and raised C-reactive protein. Haemoglobin, urea, electrolytes, liver function tests and amylase were all within normal range. Lactate level was mildly elevated (2.3 mmol l^–1^).

Chest radiograph: clear lung fields with no pneumoperitoneum.

Abdominal radiograph: unremarkable, with no evidence of dilatation of bowel.

CT aortic angiogram: arterial phase CT scan of the abdomen was performed to investigate possible vascular causes of the patient’s symptoms. It was initially reported as showing uncomplicated diverticulae, mild dependent atelectasis, few simple renal cysts, gallstones without evidence of cholecystitis, but no cause for the patient’s presentation.

## Treatment

As no cause was found for the acute presentation, the patient was admitted and managed conservatively with intravenous fluids and analgesia, and by withholding oral intake.

## Outcome

During the following 2 days, the patient remained stable, before developing sudden-onset sharp pain throughout his abdomen. On examination, his abdomen was rigid with guarding and severe tenderness; blood tests were largely unchanged.

**Figure 1.**
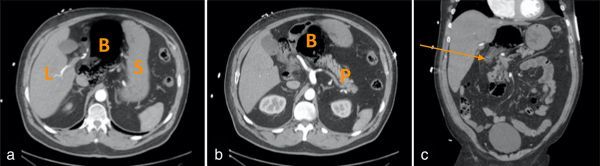
Arterial phase CT scan of the abdomen and pelvis (day 1). (a) Axial section showing hepatic flexure (B) with intraluminal faeces between the stomach (S) and liver (L), displacing the gastrohepatic ligament. (b) Axial section showing loop of hepatic flexure (B) within the lesser sac, between the pancreas (P) and the pyloric antrum. (c) Coronal section demonstrating a segment of the ascending colon passing through the epiploic foramen (arrow) into the lesser sac; the hepatic flexure is seen superior to the pancreas and medial to the stomach.

**Figure 2.**
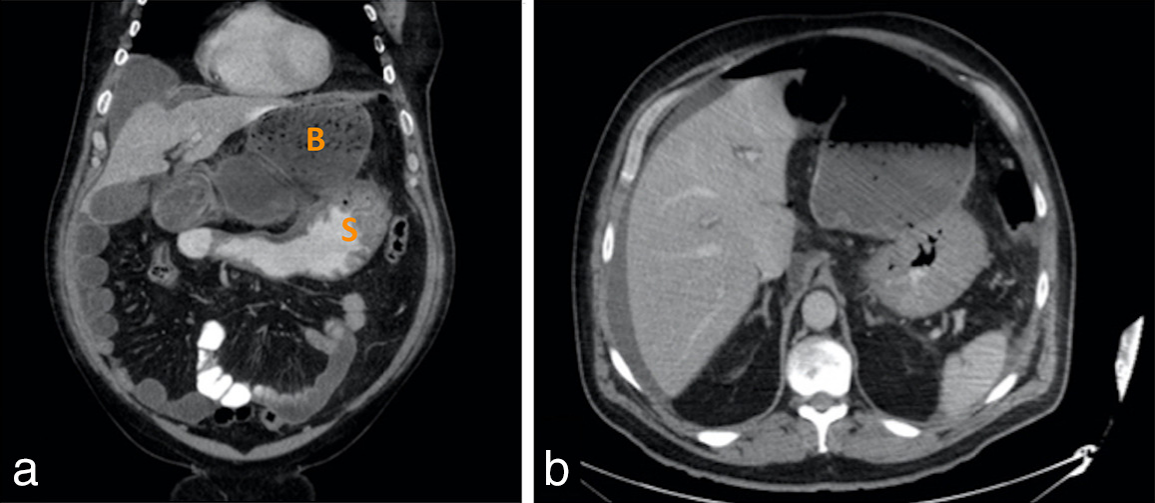
Contrast-enhanced CT scan of the abdomen and pelvis (day 3). (a) Coronal section demonstrating a grossly enlarged caecum and ascending colon (B) in the epigastrium, displacing the stomach (S) laterally and inferiorly; free fluid is also present. (b) Axial section showing the ascending colon in the lesser sac with free intra-abdominal air and fluid.

The deterioration led to re-reporting of the original images by a gastrointestinal radiologist, highlighting some subtle missed findings; a loop of hepatic flexure containing faeces and segment of ascending colon was seen between the stomach and liver (Figure 1a,b), having passed through the epiploic foramen into the lesser sac (Figure 1c). The caecum was pulled superiorly. At that point, further imaging was performed, which showed the grossly distended caecum in the upper abdomen, having further passed through the epiploic foramen. There was faecal outflow obstruction, likely owing to the distended proximal caecum and foramen itself. Free air and fluid were seen in the abdomen owing to bowel perforation (Figure 2a,b). These findings were verbally communicated to the clinical team. At laparotomy, a bascule-type caecal volvulus was confirmed and the patient underwent right hemicolectomy with primary ileocolic anastomosis. The patient made a slow recovery and was discharged 4 weeks later.

## Discussion

Lesser sac herniation is a rare occurrence, accounting for 8% of internal hernias and less than 0.1% of all abdominal hernias.^1^ Approximately two-third of lesser sac herniations contain the small bowel alone. Herniation of the large bowel, gallbladder and omentum has been reported.^2,3^ The risk factors for lesser sac herniation include: a common intestinal mesentery; ascending colon that is not attached to the parietal peritoneum; a long small bowel mesentery; and an enlarged epiploic foramen or lesser sac.^4^ In addition, increased intra-abdominal pressure was thought to play a role.^5^

Historically, lesser sac herniation has largely been diagnosed during surgery, but diagnosis is possible using imaging. On plain radiographs, the gastric bubble is displaced laterally and anteriorly by a lesser sac mass. On CT scanning, the following features have been described:^6,7^ mesenteric fat and vessesls posterior to the portal vein, common bile duct and hepatic artery; an air–fluid collection in the lesser sac, with “beaking” directed towards the epiploic foramen; two or more bowel loops in the high subhepatic spaces; and absence of the caecum from its normal anatomical position. An important differential diagnosis is paraduodenal herniation.

There is no consensus on the surgical treatment of lesser sac herniation owing to its rarity but decisions are made based on surgeon preference and bowel viability. Generally, management involves urgent surgical reduction with bowel decompression and resection of any non-viable bowel.^8^ If herniation involves the caecum, a right hemicolectomy with primary ileocolic anastomosis is the preferred option to prevent future recurrence.^9^ A caecopexy is performed if the caecum appears viable.^10^

## Conclusions

Although lesser sac herniation containing the caecum is a rare phenomenon, there have been cases described in the literature. The subtle findings associated with this important condition should not be overlooked when interpreting CT scans in patients with symptoms and signs of an acute abdomen.

## Learning points

1. Lesser sac herniation is a rare cause of bowel obstruction.
2. General radiologists should be aware of its features on CT scan.
3. Management should include urgent surgical reduction with bowel decompression and resection of non-viable bowel.
4. Re-reviewing imaging can sometimes offer further information, particularly in cases where the clinical findings do not fit the report.

## Consent

Written informed consent for the case to be published (including images, case history and data) was obtained from the patient.
